# On the Cellular and Molecular Mechanisms of Drug-Induced Gingival Overgrowth

**DOI:** 10.2174/1874210601711010420

**Published:** 2017-07-31

**Authors:** Albert Ramírez-Rámiz, Lluís Brunet-LLobet, Eduard Lahor-Soler, Jaume Miranda-Rius

**Affiliations:** 1 Department of Odontostomatology. Faculty of Medicine and Health Sciences. University of Barcelona, Barcelona, Spain.; 2 Department of Dentistry. Hospital Universitari Sant Joan de Déu. University of Barcelona, Barcelona, Spain.

**Keywords:** Fibroblast, Collagen, Drug-induced gingival overgrowth, Growth factor, Cellular cultures

## Abstract

**Introduction:**

Gingival overgrowth has been linked to multiple factors such as adverse drug effects, inflammation, neoplastic processes, and hereditary gingival fibromatosis. Drug-induced gingival overgrowth is a well-established adverse event. In early stages, this gingival enlargement is usually located in the area of the interdental papilla. Histologically, there is an increase in the different components of the extracellular matrix.

**Objective:**

The aim of this manuscript is to describe and analyze the different cellular and molecular agents involved in the pathogenesis of Drug-induced gingival overgrowth.

**Method:**

A literature search of the MEDLINE/PubMed database was conducted to identify the mechanisms involved in the process of drug-induced gingival overgrowth, with the assistance of a research librarian. We present several causal hypotheses and discuss the advances in the understanding of the mechanisms that trigger this gingival alteration.

**Results:**

*In vitro* studies have revealed phenotypic cellular changes in keratinocytes and fibroblasts and an increase of the extracellular matrix with collagen and glycosaminoglycans. Drug-induced gingival overgrowth confirms the key role of collagenase and integrins, membrane receptors present in the fibroblasts, due to their involvement in the catabolism of collagen. The three drug categories implicated: calcineuron inhibitors (immunosuppressant drugs), calcium channel blocking agents and anticonvulsant drugs appear to present a multifactorial pathogenesis with a common molecular action: the blockage of the cell membrane in the Ca2+/Na+ ion flow. The alteration of the uptake of cellular folic acid, which depends on the regulated channels of active cationic transport and on passive diffusion, results in a dysfunctional degradation of the connective tissue. Certain intermediate molecules such as cytokines and prostaglandins play a role in this pathological mechanism. The concomitant inflammatory factor encourages the appearance of fibroblasts, which leads to gingival fibrosis. Susceptibility to gingival overgrowth in some fibroblast subpopulations is due to phenotypic variability and genetic polymorphism, as shown by the increase in the synthesis of molecules related to the response of the gingival tissue to inducing drugs. The authors present a diagram depicting various mechanisms involved in the pathogenesis of drug-induced gingival overgrowth.

**Conclusion:**

Individual predisposition, tissue inflammation, and molecular changes in response to the inducing drug favor the clinical manifestation of gingival overgrowth.

## 1 INTRODUCTION

**Fig. (1):**
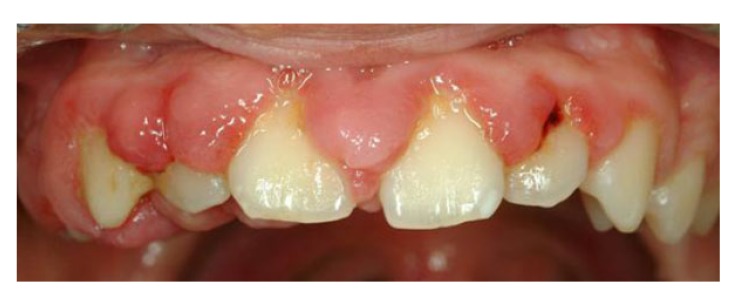
Clinical image. Gingival enlargement induced by drugs.

Gingival overgrowth (GO) has been linked to multiple factors such as adverse drug effects, inflammation, neoplastic processes, and hereditary gingival fibromatosis. Drugs associated with this gingival alteration include some antiepileptic medications, calcium channel blockers, and immunosuppressors [1-6]. The prevalence of drug-induced gingival overgrowth (DIGO) is estimated to be 70% in the case of phenytoin and 30% for other anticonvulsant drugs [7], 30% for nifedipine [8], 30% for diltiazem [9], 20% for verapamil [9], and 50-80% for cyclosporine [10, 11]. Clinical features of DIGO, such as its appearance and localization, are similar for all inducing drugs. GO usually develops during the first 3 months and reaches a plateau phase at 9 to 12 months. In the initial stages, it appears as a localized nodular enlargement of the interdental papilla (horizontal growth) and, with further progression, extends to the dental crown (vertical growth). In severe cases, the overall volume increase may cover a large portion of the clinical crown. (Fig. **1**) The changes in gingival contour may be exacerbated by plaque-induced inflammation, leading to edematous and hyperemic gingiva and thus perpetuating the cycle of GO. Local irritant factors such as cervical caries, overhanging dental restorations, food impaction, and abnormal relationships between adjacent and antagonist teeth may aggravate the severity of the condition. Its greatest prevalence is observed in children and adolescents [11-15].

The histopathological characteristics of all forms of DIGO are similar. In general, the condition presents a parakeratinized squamous epithelium with acanthosis and elongated rete pegs extending deep into the connective tissue. The lamina propria shows collagen fibrosis with variable fibroblasts, an increase of vascularity, infiltration of inflammatory cells containing plasma cells and lymphocytes, and an amorphous ground substance with evident changes of glycosaminoglycans (GAGs) [4, 16-18].

According to some authors, the onset of DIGO in the interdental papilla could be due to certain molecular histologic characteristics specific to this zone. As a consequence, a number of measurement indices focused on this area have been developed [13, 19].

The myofibroblast cell possesses profibrotic characteristics capable of synthesizing molecule compounds for tissue remodeling as observed in DIGO. Its presence is a well-established feature of the wound healing process, specifically at the transition from granulation tissue to the remodeling phase, which suggests that normal tissue turnover is exacerbated by these drugs. Additional data have provided evidence of the similarity between wound repair and fibrotic disease. Immunohistochemistry studies have identified growth factors (GF) and cytokines that regulate interactions between cells and the extracellular matrix (ECM). These polypeptide molecules are secreted by macrophages, lymphocytes, mast cells, and fibroblasts which play a key role in the inflammatory response [4, 16, 20].

The DIGO pathogenic mechanisms comprise the presence of genetically predetermined gingival fibroblasts – *responders –* that are more sensitive to the GO-inducing drug than other fibroblast subpopulations. Such fibroblast heterogeneity presents variable behavior in the production of potentially proliferative, fibroblastic cytokines/GFs and their environmental response related to ECM components [21].

The fibrotic clinical appearance of DIGO is due to a disproportion in the synthesis-degradation of collagen. The most genetically-sensitive fibroblast, in the presence of inducing drugs, synthesizes the greatest quantity of collagen [22-24] and reduces collagenolytic activity, with a slowing-down of its catabolism by collagenase action [24-28], or increases collagen deposits by inhibiting endocytosis [26, 29-32].

Connective tissue is present in many structures and organs. It is not clear, however, why gingival fibroblasts react so specifically to GO-inducing drugs. A few authors have reported cases of cutaneous alterations or a greater incidence of keloid scarring in patients treated with these drugs, or an increase in the incidence of illnesses related to the fibrosis of other organs and systems. It would be interesting to examine this issue in greater depth, since gum alterations vary so widely in populations treated with similar GO-inducing drug dosage, and also because the alterations rarely appear at the systemic level [33]. Moreover, this morphological alteration does not affect the different areas of the buccal cavity and the gingival tissue in the same way. Research into this gingival pathology should be continued as it could shed light on the etiopathogeny of other illnesses related to collagen and connective tissue.

The main objective of this literature review is to analyze the cellular and molecular mechanisms related to the pathogenesis of pharmacological gingival overgrowth. We present several causal hypotheses and discuss the advances in the understanding of the mechanisms that trigger this gingival alteration.

## 2 CELLULAR AND MOLECULAR BIOLOGY OF GINGIVAL OVERGROWTH

DIGO is a specific morphologically conditioned alteration whose fibrotic process does not usually affect other systemic locations in the same manner [33]. The gingiva is a tissue exposed to various detrimental factors such as bacterial biofilm, brushing, and masticatory microtrauma; also its influenced by various factors including dehydration, due to labial incompetence or mouth breathing, and a limited anterior interdental space [13]. Consequently, it undergoes a continuous reparative process which, due to local characteristics, may lead to the onset of a fibrogenic process.

### 2.1 Interaction of Chemotactic Factors

The gingival tissue is subjected to multiple aggressions that induce a state of permanent tissue repair involving the inflammatory cells, fibroblasts and chemotactic factors. Many of these chemicals are polypeptide molecules, GF and cytokines, secreted locally by various cells in the gingiva, which regulate processes such as GO development. Basic fibroblast GF (bFGF) is a fibroblast and keratinocyte mitogen molecule with morphogenesis and differentiation functions related to fibroblastic proliferation in GO [34]. Vascular endothelial GF (VEGF) promotes endothelial cell proliferation and differentiation, induces microvascular hyperpermeability, and participates in ECM remodeling [35]. Cell migration is an important phenomenon in tissue formation and remodeling, and is controlled especially by epidermal GF (EGF) [36, 37]. Together with platelet-derived GF (PDGF) it facilitates wound healing and enhances wound strength, by the migration of macrophages and fibroblasts and the synthesis of matrix proteins [38]. Additionally, PDGF can stimulate the synthesis of other GFs whose functions are partly mediated by the induction of endogenous GF such as insulin-like GF (IGF). IGF causes an increase in fibroblast collagen synthesis and it may be relevant to the stimulation of mesenchymal tissues during periodontal regeneration [39]. Studies in samples of drug-induced overgrowth have demonstrated high concentrations of GF, among them connective tissue GF (CTGF) and the cytokines IL-6, IL-1β [40]. CTGF potentiates the growth of fibroblasts through a mechanism related to angiogenesis during injury and development [41].

The clinical and pathological features of DIGO are independent of the drug administered, which suggests a common pathway of induction in spite of the differences [9]. In a pathological environment such as DIGO, the deregulated cytokine imbalance may make a considerable contribution to its development. This intermediary molecular action reinforces the idea that the direct action of the inducing drugs is not the most important mechanism in GO development [40].

Clinically, the interdental papilla presents greater sensitivity to overgrowth than other gingival locations due to a number of specific molecular compounds involved in the wound healing process, such as surface receptors, procollagen type I, fibronectin, GAGs, and GFs [19]. As noted above, the interdental papilla area has a greater predisposition to develop GO than the marginal gingiva. DIGO appears to be linked to an up-regulation of several integrins which are highly expressed in the epithelial basal layer, especially in the papilla [42].

Proteoglycans (fibromodulin, lumican and biglycan), CTGF, type ανβ6 integrin, and the son-of-sevenless gene (SOS)-1 are more highly expressed in the papilla epithelium than in the oral epithelium of the marginal gingiva. Integrins, transmembrane receptors, are the principle mediators between the fibroblast and its ECM environment, regulating collagen fibers at the cell membrane [43].

A number of biochemical studies have shown a mutation in SOS-1, a constitutively active molecule, related to hereditary gingival fibromatosis, a type of GO. Likewise, DIGO may present a similar mutation affected by gene-encoding alteration. The discovery of significant SOS-1 immunostaining in the interdental papilla, compared to that found in the marginal gingiva, could represent a potential prosynthetic tissue [19, 44].

The tissue characteristics of the papilla favor the action of inducing drugs, which lead to its augmentation by joining the epithelium with the connective tissue and vice versa [19].

### 2.2 Genetic Variability in Cell Populations

In gingival connective tissue, fibroblasts are by far the most numerous stromal cell types. However, the fibroblast heterogeneous genotype appears to manifest different phenotype subpopulations depending on anatomical location and the single site of the periodontium. These cells are genetically determined to present different characteristics during cell replication, molecular secretion, cytoskeletal proteins, affinity in receptor bonding, and secretion of matrix-degrading enzymes [21, 45]. In granulation tissue, the fibroblasts acquire the morphological and biochemical features of smooth-muscle cells by expression of α-smooth muscle actin. The subtype, the myofibroblast, is active during the reparative process of DIGO, but disappears selectively due to apoptosis and the survival of other fibroblast groups. Myofibroblast apoptosis is critical for normal healing as the persistence of this cell favors continuous ECM deposition and the development of pathologic fibrotic conditions [46]. Variation in enzyme behavior is particularly noticeable in the collagenases which take part in collagen metabolism for tissue remodeling in a number of processes including turnover in wound healing. Collagenases belong to a larger family named matrix-remodeling metalloproteinase (MMP). Collectively, they can process bioactive molecules and degrade several extracellular matrix proteins. They are all synthesized in a latent form (for example, a proenzyme). In contrast, tissue inhibitor of MMP (TIMP) enzymes hinder MMPs; their relationship is modulated by the heterogenic collagenolytic response which is halted in fibroblast cultures induced by cyclosporine [47].

Moreover, fibroblast heterogeneity may lead to an imbalance of cells that produce excessive collagen and do not effectively remodel the nascent ECM. Fibroblast heterogeneity, influenced by the regulatory role of cytokines and GF, results in a heterogeneous remodeling response to drug injury. In a similar manner, other epithelial phenotypes may be conditioned by interactions with the connective tissue below. The localized specificity of the epithelium is influenced in part according to the phenotypes of underlying fibroblasts [21].

Several Human Leukocyte Antigen (HLA) phenotype combinations are involved in the development of gingival alteration in morphology. Some organ transplant recipients, under medical treatment with cyclosporine, present conspicuous clinical GO significantly associated with the specific haplotype gene expressions [15, 48].

Lymphocyte and macrophage populations of gingival tissue affected by inducing drugs also manifest varying sensitivity in their inflammatory action, and differ from those in healthy gingival controls [49, 50].

The presence of genetic polymorphisms is also related to the action of DIGO linked to diverse molecular compounds. The phenotypical manifestation of the multidrug resistant (MDR1) gene has been observed, which determines drug resistance capacity in the p-glycoprotein molecule for cyclosporine [51-53]. An additional polymorphism with respect to membrane integrin receptors has been reported in a number of studies. Some authors have related α_2_ integrin polymorphism in the bonding capacity of collagen to the cell surface which might modify collagen phagocytosis [43]. Studies of its association with calcium channel blocker-induced GO confirm that it could be genetic risk factor for DIGO [54].

### 2.3 The Extracellular Matrix: Increases in Collagen and Glycosaminoglycan Concentrations

Fibrosis in gingival tissue is one of the main causes of DIGO. The action of inducing drugs on cell mechanisms affects the intra-cellular calcium, reducing the cationic cell influx due to changes in the sodium-calcium exchange [26]. Calcium acts as a second messenger at an intracellular level. Its regulation depends on mechanisms that modulate cell membrane flux and its release from intracellular deposits. Calcium bonds with proteins and activates target molecules, for instance enzymes, and target such as ionic channels. It also modulates cellular transcription and proliferation, and functions related to the ECM through the integrins [44].

The maturation of collagen and elastin in the ECM depends on post-translational modifications, including the partial regulation effected by lysyl oxidase, a copper-dependent extracellular enzyme produced by fibrogenic cells that catalyzes the extracellular oxidative deamination of peptidyl-lysine and peptidyl-hydroxylysine residues of tropocollagens. The mature collagen in its covalent bonds is insoluble in the ECM and is deposited, thus reducing the turnover [55].

Transforming GF Beta (TGF-β) action is linked to inducing drugs, through enzymatic reactions such as lysyl oxidase activity and the inhibition of the collagenase. It stimulates the fibroblastic population and the ECM deposit of fibronectin and GAGs [56, 57]. Fibronectin is a non-fibrous protein that facilitates cell adhesion to the matrix. The integrin protein mediates its link with actin filaments on the fibroblast surface for cell motility, and regulates gene expression, cell proliferation, and cell migration [57].

Inducing drugs act through the regulatory effects of GF and cytokines, not directly on the activity of lysyl oxidase or at the transcriptional level [55]. TGF-β is believed to be a key mediator of tissue fibrosis for ECM accumulation in some pathologic states such as progressive renal diseases due to cyclosporine-induced nephropathy. It has been observed that gingival fibroblasts which are overgrown due to cyclosporine show a high fibroblast proliferative capacity through this GF, with increased collagen synthesis and a reduction of secreted active collagenase levels [23, 47, 58, 59]. TGF-β enhances neovascularization *in vivo* by stimulating the cells to secrete angiogenetic factors [58, 59]. Phenytoin and nifedipine have also been reported to increase collagen synthesis in some *in vitro* studies [22, 24].

CTGF regulates the proliferation-differentiation of connective tissue cells stimulating ECM production. When CTGF binds with integrin α_6_β_1_ an insoluble collagen accumulation appears in gingival human fibroblast cultures and eventually stimulates ECM production. CTGF may also play a role in angiogenesis through its association with vascular endothelial cells, and is highly secreted in tissue microenvironments induced by phenytoin [41]. It appears that certain intrinsic links between these molecules may potentiate or inhibit their combined activity. Some authors have reported that the binding between CTGF and TGF-β may become a mediator, which reinforces the fibrogenic function of the fibroblast [40, 41, 60]. TGF-β and CTGF potentiate the activity of lysyl oxidase, unlike PDGF, IL-1β, and IL-6 [55].

Fibroblast collagen phagocytosis is the regular catabolism for ECM degradation. It is well known that this process is related to some biochemical modifications at the integrin receptors when collagen adheres to the membrane cell. A reduction in the expression of integrin α_2_ by inducing drugs, or a decrease in collagen adhesion due to the presence of pro-inflammatory cytokines, could inhibit phagocytosis and give rise to the development of GO [26, 29, 31, 32, 43, 61, 62].

Other surface molecules, discoidin domain receptors (DDRs), are activated in the presence of collagen and regulate β1integrin receptors. It has been shown that cyclosporine may also affect DDRs and reduce β1 integrin, which allows focal adhesion of collagen at the onset of its phagocytosis [62].

Cathepsins are lysosomal cysteine enzymes which catalyze collagen internalization and metabolism through hydrolysis cascade reactions. Some cathepsins are able to degrade fibrinogen, the basal membrane, and fibronectin and activate latent collagenases, thus indirectly regulating ECM metabolism. Phenytoin, cyclosporine, and nifedipine suppress cathepsin action, reducing protein metabolism in the ECM and at the level of their transcription [30, 63].

Collagen degradation also occurs through the secretion of collagenases in the extracellular pathway. The activation of collagenase is a complicated process which depends upon multiple biochemical pathways. The production of the active form (collagenase-activating enzyme) is additionally limited when the calcium influx decreases and simultaneously reduces cellular folic acid uptake, a process favored by inducing drugs. Decreased cellular folic acid leads to increase TIMP-1 which reduces MMP-1; as MMP-1 is necessary for the activation of collagenase, the result is a reduction in the amount of activated collagenase. It appears that folic acid may be of benefit in preventing the recurrence of phenytoin-induced gingival changes which initiate gingival fibrosis. Also, in the presence of inflammation secondary to dental plaque, the catabolic ability of collagenase is saturated, and the inhibited degradation of the ECM causes a local accumulation of this matrix [1, 27, 28, 31, 32, 47,64].

Fibroblasts modify the biochemical composition of the ECM at the collagen fibers, thus increasing the total quantity of GAGs and modulating the immune system and inflammatory response [65]. This process is largely mediated by GF due to an imbalance in the proportion of TGF- α β, bFGF, PDGF, and CTGF and other biological molecules. Conversely, the ECM modulates cell function in the interaction among proteoglycans, several cytokines (IL-1, 6) and enzymes [34, 38].

Hyaluronan is a non-sulfated GAG present in the ECM providing structural support to cells. Intense TGF-β output, such as the promotion of GAG synthesis under the influence of cyclosporine and nifedipine, has been observed in gingival fibroblast cultures [56, 57].

Cyclosporine A could induce an up-regulation of some heparin sulfate proteoglycans, on occasions involving the PDGF mediator [23, 38, 65, 66]. The combined action of two inducing drugs, such as nifedipine and cyclosporine, on fibroblast cultures allows a greater incorporation of sulfates to the GAGs than individually [67]. In a study using phenytoin, the authors described differences depending on the anatomical location in the gingiva: intracellular sulfated GAGs in attached gingiva and extracellular sulfated GAGs in free gingiva [45]. These findings for proteoglycans may provide evidence that the non-collagenous extracellular matrix in DIGO is overexpressed.

### 2.4 Fibroblast Proliferation Versus Apoptosis

Several studies have reported different effects of inducing drugs on cell proliferative activity and their relationship with apoptosis in diverse populations in the gingival tissues. Apoptosis is a type of genetically determined cell death necessary for tissue regulation. These differences are due in particular to the genetic variability of the cellular elements with phenotypes of different behavior [68].

Fibroblast viability has been analyzed in the presence of nifedipine and cyclosporine with a clearly increased replication of these connective cells [22, 59]. In a similar manner, an *in vitro* study of phenytoin and nifedipine-induced GO demonstrated a high presence of bFGF in the fibroblast culture which would confirm hyperplasic cell growth [34]. On the other hand, a study regarding primary cultures of human gingival fibroblasts with the presence of cyclosporine, nifedipine, and phenytoin did not find significant changes in drug induction depending on the length and the dosage of treatment [69]. In the same study, mRNA expressions of the collagen, TGF-β, and collagenase proteins were observed to be significantly increased for some doses. However, the authors reported an unexpected increase in mRNA collagenase which, due to a disruptive protein translation, may have remained in an inactive collagenase form or in saturated form following inflammation caused by bacterial plaque [24, 25].

Apoptosis is analyzed *in vitro* with index-molecules associated with cell death in fibroblast populations in cultures. The following molecules are considered specific for cell destruction: the caspase-3 enzyme, secreted by many cell types, and the mitochondrial protein markers BCL-2 (which expresses a reduction in apoptosis) and Bax (which reflects an increase). Some studies report a reduction in apoptosis: decreased levels of caspase-3, upregulated anti-apoptotic BCL-2 and downregulated pro-apoptotic Bax [70, 71]. Studies of fibroblast cultures have shown that calcium channel blockers could significantly inhibit the adherence-induced death of fibroblasts after cell confluence [72]. The combined induction of LPS and cyclosporine in the fibroblast cultures produced a reverse effect on the LPS inhibition of cell proliferation [73]. The same action has been observed when PDGF is able to reverse the inhibitory LPS action on fibroblast proliferation and fibroblast synthetic activity in gingival cell cultures [74].

Apoptosis has been shown to be modulated by pro-inflammatory mediators. It appears that some cytokines such as Tumor Necrosis Factor alfa (TNF-α) stimulate the expression of transcription factors related to the gene expression of apoptosis. These factors balance the relationship between proliferation and apoptosis [71].

The experimental results in keratinocyte populations reflect the same diversity. Various authors have shown that cyclosporine-induced GO could be related to an increase of keratinocyte life span and not to a greater keratinocyte proliferation since it seems probable that some inducing drugs, such as cyclosporine and nifedipine, could inhibit apoptosis regulation in the gingival epithelium [71, 75, 76].

In contrast, other authors have found an increase in the proliferative capacity of keratinocytes due to inducing-drugs in samples of GO with increases in the marker Ki67, typical of cell proliferation, and an elevated bFGF type or keratinocyte growth factor (KGF) concentration in the gingival tissue with high mitotic activity [34, 76, 77- 79]. It has been observed that the proliferation rate of epithelial cells could be linked to the interaction of fibroblasts [18]. Such a relationship may depend on the sensitivity of the fibroblast subpopulations to the inducing drugs.

### 2.5 Oxidative Stress and Intermediate Compounds

Nitric oxide (NO) is a cellular signaling molecule with vasodilatory function, synthesized in the endothelium by enzymatic activity of nitric oxide synthase (NOS), sometimes promoted by the endothelin-specific receptor [80]. Occasionally NO is also produced in the inflammatory environment by an inducible form of nitric oxide synthase (iNOS) from resident inflammatory cells [81]. Moreover, the LPS and other specific cytokines such as IL-1, TNF-α, and interferon-γ may increase iNOS synthesis [71, 72]. Some authors have reported that iNOS is highly expressed in epithelium and connective tissues in gingivitis, and up-regulated in cyclosporine-induced GO with regard to healthy gum [81]. It has been observed that the vascular endothelium responds to reactive oxygen species (ROS) arising from oxidative stress, which appears in metabolic situations of imbalance between ROS and cellular antioxidant defense mechanisms, thus increasing iNOS activity [82].

Synthesized NO, as a free radical, may react with several ROS in a cellular aerobic metabolism condition to produce nitrating compounds: some of them, like peroxynitrate, highly oxidizing. A recent study found these compounds in the saliva of phenytoin responders in significantly higher quantities than in the groups induced by other drugs. There appears to be a significant correlation of salivary nitrite concentration with periodontal parameters such as probing depth, gingival bleeding time index, severity of GO, and GCF volume. Thus, salivary nitrites could be used as biomarkers in phenytoin-induced GO [83].

Additionally, ROS play a critical role in oxidative stress which seems linked to the initiation and progression of fibrotic diseases [82, 84]. In the same way, it has been observed that cyclosporine could induce intracellular ROS generation and eventually stabilize the ECM structure by increasing its resistance to protease degradation [82].

According to several studies, mast cell proteases may be involved in DIGO and in some tissue repair mechanisms. It appears that these released proteases encourage fibroblastic replication, and increase collagen synthesis and ECM formation [85]. Chymase, one of these secreted enzymes, may also participate in the formation of angiotensin II, an intermediate compound in the renin-angiotensin homeostatic system, with pro-inflammatory, pro-coagulant and pro-fibrotic capacity [86]. Furthermore, research in fibroblast cultures has demonstrated a drug-induced inhibition of calcium influx which could act as a stimulatory signal increasing angiotensin II and endothelin-1 protein vascular mediators and might have profibrotic effects [80, 87]. Although cyclosporine action may diminish mast cell number it can also modulate the gingival expression of angiotensin II [88]. In gingival samples from healthy subjects and patients receiving nifedipine, significantly elevated levels of angiotensin II were observed in nifedipine *responders* compared with healthy individuals and patients on nifedipine without GO [86].

### 2.6 Immunity Changes and Inflammation

Inducing drugs may produce a reparative process controlled by inflammation which promotes a chronic accentuated fibrotic response in the ECM. DIGO secondary inflammation could be necessary to trigger the onset of the fibrotic process, but not for its progression [89]. Access to bacterial biofilm in gingival tissue as well as physical insults mean that the inflammatory process is responsible for the severity of DIGO, as several studies have concluded [90].

Immune-inflammatory features associated with DIGO include increased macrophage reparative/proliferative phenotype, up-regulation of essential GF, IL-1β, and IL-6 cytokines, and variable lymphocyte proportions [38, 49, 91-93]. Several studies have described a lymphocyte infiltration of plasma cells in DIGO samples. This would suggest that the humoral immune response replaces the cellular immune response in cyclosporine samples due to its immunosuppressive action. Moreover, the pattern in patients with cyclosporine-induced GO is characterized by the low expression of some types of lymphocytes (natural killer lymphocytes) in contrast to chronic inflammatory periodontal disease [50]. Histologically speaking, it is important to note the proportions of fibrosis and inflammation with respect to the drug used: phenytoin causes moderate inflammation and high fibrosis, nifedipine produces moderate inflammation and fibrosis, whilst cyclosporine leads to intense inflammation and low fibrosis [40]. It is possible that gingival enlargement produced by cyclosporine presents a very pronounced immune response with some moderate antifibrotic effects in the synthesis and deposition of collagen [90].

One of the main mechanisms of inducing drugs is participation in the synthesis cascade of prostaglandins (PGs). Phenytoin stimulates PG precursors, such as arachidonic acid, and enhances phospholipase activity [94]. According to *in vitro* and *in vivo* culture studies, cyclosporine could inhibit cyclo-oxygenase 2 and consequently PGI_2_ mediated polypeptide compounds [95, 96].

PGs and thromboxane molecules, which are synthesized at the onset of the inflammatory process, interact with a number of molecules related to collagen metabolism in the ECM such as pro-inflammatory cytokines and GF. IL-1β is the main inflammatory mediator in the pathogenesis of periodontal disease; it promotes MMP production with other cytokines, and activates T cells and neutrophils. In addition, IL-1β potentiates bone resorption through osteoclast formation [97].

Once the PG and thromboxane cascade is activated, deregulated pro-inflammatory cytokines could appear from the immunomodulatory effects of the inducing drugs and stimulate a down-regulation of MMP [28, 98].

Under drug-inducing treatment, the inflammatory cellular profile may present a modification in the proportion of macrophage phenotypes (inflammatory, reparative, or resident), variable lymphocyte subpopulations, and their distribution in peripheral blood, in contrast to healthy control gingival tissues [16, 38, 40, 49, 92].

### 2.7 Other Predisposing Factors

#### 2.7.1 Dental Plaque

Bacterial plaque located in the gingival sulcus epithelium is an aggravating factor in GO. Clinically, the interdental areas present the highest plaque index, which coincides with the greatest GO. The significant correlation between the two factors confirms the hypothesis of the predisposing role of plaque in GO development, whilst GO simultaneously favors dental plaque accumulation in the pseudopockets. Some authors state that the elimination of plaque is a preventive measure for drug-induced GO [10, 12]. There is a high cell turnover of keratinocytes, particularly in the junctional epithelium, and gingival fibroblasts with inherent heterogenic phenotype populations that have a pronounced differential capacity [16, 21, 77].

#### 2.7.2 Dental/Periodontal Microtrauma

Some authors have reported that the combined presence of mechanical stress and active TGF-β1 is essential to convert fibroblasts into contractile myofibroblasts, which cause tissue contractures in fibrotic diseases [99]. These mechanical forces are decisive in gingival tissue development and its reparative process. On the fibroblast membrane surface there is frequent exposure to pressure and traction due to mechanical forces, especially in the presence of occlusal microtrauma or during orthodontic movement, which produce stress in the gingival keratinocytes and fibroblasts. Studies have reported that EGF is involved in fibroblast motility and fibroblastic migration processes [37]. Physical signals are transmitted to the fibroblast surroundings through the integrin receptors, which could stimulate the synthesis of ECM constituents such as collagen and also regulate the inflammatory response in reparative processes [23, 24, 36].

Traditionally, the appearance of gingival enlargement in patients during orthodontic treatment was attributed to the accumulation of bacterial plaque. However, some patients with adequate bacterial plaque control may present gingival overgrowth without signs of inflammation [100].

Surlin *et al.* hypothesize that gingival enlargement following orthodontic treatment is not always associated with signs of inflammation but may be a response to mechanical stress and periodontal remodeling during orthodontic tooth movement. MMP8 levels in gingival crevicular fluid in patients wearing fixed braces can be considered as indicators of this process [101, 102].

## 3 DISCUSSION

**Fig. (2):**
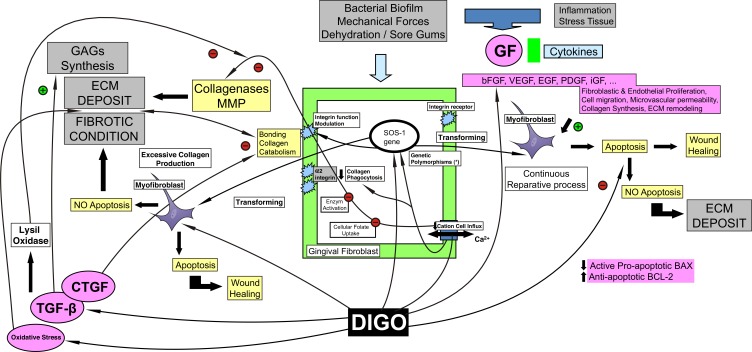
Diagram Summary. The figure shows a simplified schematic model, which is applicable to all the inducing drugs, although the predominance of the pathogenic mechanisms described may vary.The gingival tissue is subjected to multiple aggressions that induce a state of permanent tissue repair involving the inflammatory cells, fibroblasts and chemotactic factors. The presence of myofibroblasts is a well-established feature of the wound healing process.Genetically predetermined gingival fibroblasts *-responders-* are more sensitive to the GO-inducing drugs than other fibroblast subpopulations. This fibroblast heterogeneity leads to variations in the production of potentially proliferative, fibroblastic cytokines/GFs, and in their environmental responses to ECM components; myofibroblast cell possesses profibrotic characteristics capable of synthesizing molecule compounds for tissue remodeling, as observed in DIGO. The myofibroblast is active during the reparative process of DIGO, but disappears selectively due to apoptosis and the survival of other fibroblasts groups. Myofibroblast apoptosis is critical for normal healing, as the persistence of cell favors continuous ECM deposition and the development of pathologic fibrotic conditionsDIGO – Drug Induced Gingival OvergrowthSOS-1 – Genetic variability of fibroblasts with phenotypes of different behavior (Integrin function modulation)(*) Genetic risk factor for DIGO.

This review article presents a wide-ranging investigation of the literature. It discusses the various agents involved in the pathogenesis of DIGO, assesses specific aspects related to them, and presents a general hypothesis based on an original diagram that may help to understand the process (Fig. **2**).

The adverse effect of DIGO appears to be produced by three different drug categories: calcineurin inhibitors (immunosuppressant drugs), calcium channel blocking agents, and anticonvulsant drugs, which all share the same pathogenic mechanism. All the drugs associated with GO appear to alter the cation inflow in the cell membrane (Na^+^ and Ca^2+^ channels). The alteration of the uptake of cellular folic acid, which depends on the regulated channels of active cation transport and on passive diffusion, results in a dysfunctional degradation of the connective tissue [64].

In an epidemiologic sense, GO-inducing drugs have individually shown varying clinical impacts. Combined pharmacological therapies have occasionally been reported to increase GO prevalence and severity [48, 103]. DIGO severity could be related to dosages and pharmacologic plasma levels [11, 104], although most authors have reported no correlation [53, 105, 106]; nor has any relationship been found between TGF-β gingival concentration and TGF-β plasma levels in DIGO cases. Nevertheless, this GF is considered to be a risk factor for GO due to the increase of gingival levels [107, 108].

The issue of gingival fibroblast heterogeneity is interesting, and finding a related gene in the three drug categories is particularly challenging. However, it is rather difficult from these data to determine the mechanism of the side-effect. In the future, if the related gene is also discovered, it may be possible to draw particular inferences regarding the pathogenesis.

It may also be that the direct action of the drug on the fibroblast is not sufficient, and that the intermediary molecular action/deregulated cytokine imbalance, may make a considerable contribution to the development of DIGO. Inflammatory effects have been described due to changes in cell and lymphocytic infiltrate with the release of growth factors, cytokines and prostaglandins, which influence connective tissue homeostasis and the composition of glycosaminoglycans in the extracellular matrix. The three groups of drugs act by affecting the collagen turnover, with changes in the synthesis and in the collagenolytic action which increase the fibrotic deposition in the extracellular matrix.

Clinically it is clear that patients with poor plaque control will have more severe GO due to the effects of these inducing drugs. Normally the inflammation favors the activation of a repair process until the tissues heal. However, phenytoin,nifedipine and cyclosporin A may interfere in this physiological process, as noted above.

## CONCLUSION

A number of different cellular and molecular mechanisms in the epithelial and connective tissue are involved in the pathogenesis of drug-induced gingival overgrowth. Cytokines and growth factors play a crucial role in developing this tissue imbalance. In most cases, inflammation and fibrosis are the main concomitant processes related to this gingival alteration. Occasionally other systemic intermediary molecules may interact in this process. Some specific anatomical areas such as interdental papilla have the greatest risk of gingival overgrowth. The development of pharmacological gingival overgrowth is subject to genetic susceptibility. Further studies are required for a greater understanding of the interaction of medication and gingival tissue cells for the prevention and treatment of this periodontal pathology.

## LIST OF ABBREVIATIONS

DIGO: = drug-induced gingival overgrowth;
ECM: = extracellular matrix;
GO: = gingival overgrowth;
GAGs: = glycosaminoglycans;
GF: = growth factors;
bFGF: = basic fibroblast GF;
VEGF: = vascular endothelial GF;
EGF: = epidermal GF;
PDGF: = platelet-derived GF;
IGF: = insulin-like GF;
CTGF: = connective tissue GF;
SOS: = son-of-sevenless gene;
MMP: = matrix-remodeling metalloproteinase;
TIMP: = tissue inhibitor of MMP;
HLA: = human leukocyte antigen;
MDR1: = multidrug resistant;
TGF-β: = transforming GF Beta;
DDRs: = discoidin domain receptors;
TNF-α: = tumor necrosis factor alfa;
KGF: = keratinocyte growth factor;
NO: = nitric oxide;
NOS: = nitric oxide synthase;
iNOS: = inducible form of nitric oxide synthase;
ROS: = reactive oxygen species;
PGs: = prostaglandins
